# The risk and severity of stroke in patients with atrial fibrillation and gout: A National Representative Database study

**DOI:** 10.1002/joa3.12505

**Published:** 2021-01-25

**Authors:** Faris Haddadin, Ana B. Arevalo, Ahmad Jabri, Hassan Beydoun, Farah Fram, Alba Munoz Estrella, Saima Karim, Salim Virani, Yousaf Ali

**Affiliations:** ^1^ Section of Cardiology Baylor College of Medicine Houston TX USA; ^2^ Department of Medicine Icahn School of Medicine at Mount Sinai Morningside New York NY USA; ^3^ Heart and Vascular Center Metrohealth Medical Center Case Western Reserve University Cleveland OH USA; ^4^ Department of Cardiology University of Arizona COM‐Phoenix Phoenix AZ USA; ^5^ Faculty of Medicine University of Jordan Amman Jordan; ^6^ Mount Sinai Heart Icahn School of Medicine at Mount Sinai Morningside NY USA; ^7^ Section of Cardiology Michael E. DeBakey Veterans Affairs Medical Center Section of Cardiovascular Research Department of Medicine Baylor College of Medicine Houston TX USA; ^8^ Department of Rheumatology The Mount Sinai Hospital New York NY USA

**Keywords:** atrial fibrillation, gout, stroke

## Abstract

**Background:**

It has been shown that gout is associated with left atrium remodeling and a pro‐inflammatory state leading to the development of atrial fibrillation (AF). There is limited evidence whether gout increases the risk of stroke in patients with AF. We assessed the incidence of gout and the risk of stroke in patients with AF.

**Methods:**

This is a retrospective cohort study using the 2016 US National Inpatient Sample (NIS) based on ICD‐10 codes. The outcomes of the study were the risk and severity of new stroke in patients with pre‐existing AF and gout.

**Results:**

In 2016, we identified 3 844 057 patients admitted to the hospitals in NIS with history of AF, of which 240 875 had history of gout. Patients with AF and gout have higher risk of new stroke (OR 2.07 [1.97‐2.19], *P* < .001), and this risk remains significantly elevated after adjusting for CHADS2VASC score variables, chronic kidney disease, dyslipidemia, obesity, and race (OR 1.10 [1.01‐1.11], *P* = .041). However, presence of gout in patients with AF was not associated with all‐cause in‐hospital mortality, need for mechanical ventilation, percutaneous gastrostomy tube insertion, or discharge to skilled nursing facility.

**Conclusion:**

Subjects with AF and gout compared to AF alone had an increased risk of new stroke, but presence of gout was not associated with stroke severity. There is a potential role of gout as a risk factor or a risk marker for stroke in subjects with AF.

## Graphical abstract

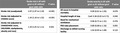

## 1 INTRODUCTION

Atrial fibrillation (AF) is the most common arrhythmia and is associated with an increased risk of stroke and heart failure.^1^ Uric acid level, a commonly elevated cellular metabolite in subjects with gout, is theorized to cause systemic inflammation, which in turn may contribute to the pathogenesis of cardiovascular disease and arrhythmias.^2^

Gout is the most prevalent inflammatory arthritis worldwide, is associated with cardiovascular and renal diseases, and is an independently associated with premature death and metabolic syndrome.^3^, ^4^ Elevated uric acid level in subjects with gout is associated with cardiovascular conditions, such as hypertension (HTN), which are considered risk factors for AF and stroke.^5^ Krishnan et al found a 9% increase in the risk for developing arterial HTN in men without metabolic syndrome with each unit increase in serum uric acid.^6^ Recent studies have shown that gout is independently associated with a higher risk of AF at diagnosis and the risk remains elevated after the diagnosis.^7^

Elevated uric acid in subjects with gout leads to proinflammatory effect and vascular inflammation which may increase the risk of atrial arrhythmias and cardiovascular disease.^2^, ^8^ However, since there are many confounding variables, the relationship between gout and AF has remained inconsistent.^9^ Whether or not presence of gout increases the risk of cerebrovascular events in those with AF remains less studied. Our study is aimed at re‐examining this relationship and the role of gout as a stroke risk factor in AF in a large inpatient North American dataset.

## 2 METHODS

### 2.1 Data source

This study was based on the National Inpatient Sample (NIS) for the year 2016. The NIS is part of all‐payer database developed and sponsored by the Agency for Healthcare Research and Quality for the Healthcare Cost and Utilization Project (HCUP). The NIS is the largest publicly available inpatient healthcare dataset in the United States and represents 20% of all discharged patients every year from both urban and rural hospitals, excluding rehabilitation centers and long‐term care facilities. All hospitalized patients are included regardless of the payer. The NIS allows analysis trend over time since 1988 and it includes 46 states. The new NIS sampling strategy produces a precise estimate of more than 97% of the US population. Patient confidentiality is protected as state and hospital identifiers are not provided. The NIS 2016 utilizes the International Classification of Diseases, Tenth Revision, Clinical Modification codes (ICD‐10‐CM). Institutional Review Board (IRB) review and approval was not required as the NIS is a publicly available dataset that contains de‐identified patient information.

### 2.2 Study population

The NIS provides as many as 30 discharge diagnosis and 15 procedures recorded by using the ICD‐10‐CM codes. All adults (older than 18 years of age) with history of atrial fibrillation (AF) in 2016 were included in the current study. Risk of new stroke and post‐stroke sequalae in patients with AF were stratified based on whether the patient had the diagnosis of gout based on prevalence measurements. The list of diagnosis ICD‐10‐CM codes used to identify patients is shown in Table S1.

### 2.3 Study variables and outcomes

We first compared the baseline variables in all patients with AF, with and without gout. Study variables included age, gender, race, HTN, obesity, diabetes mellitus (DM), congestive heart failure (CHF), dyslipidemia, coronary artery disease (CAD), peripheral vascular disease (PVD), history of stroke or transient ischemic attack (TIA), tobacco smoking, chronic kidney disease, and end stage renal disease (CKD/ESRD). We included all stages of CKD in this study. The primary outcome of the study was the risk of new stroke in patients with pre‐existing AF and gout. New stroke was identified by the ICD‐10‐CM codes with the diagnosis of cerebral infarctions due to vessel thrombosis or embolus, occlusion and stenosis of cerebral or precerebral vessels without infarction and sequelae of cerebrovascular disease as the primary admission diagnosis. The secondary outcomes were the severity and post‐stroke sequelae in patients with AF and gout. These outcomes were assessed through all‐cause in‐hospital mortality, hospital length of stay (LOS), the need for mechanical ventilation (MV), the need for percutaneous endoscopic gastrostomy tube (PEG) insertion, and discharge to a skilled nursing facility.

### 2.4 Statistical analysis

Categorical variables are presented as frequencies and percentages while continuous variables are presented as mean ± SD. Differences between groups were assessed with Pearson Chi square test for categorical variables and Student's *t*‐test for continuous variables. We assessed the association between gout and stroke in patients with AF using two models. The first was an unadjusted univariable logistic regression model and the second was a multivariable logistic regression model adjusted for potential confounding factors to calculate the odds ratio and their 95% confidence interval. We adjusted for potential confounders in two steps, the first was for the CHADS2 score variables, which includes CHF, HTN, age 75 years and older, DM and prior history of stroke or TIA. The second step of adjustment included CHADS2VASC score variables, which included CHADS2 score variables in addition to female gender, vascular disease history such as CAD and PVD, and any other significantly different baseline characteristic variable that is not accounted for in the CHADS2VASC score between patients with AF and gout who had new stroke and those with who did not (Table 2). The second step of adjustment was also used to assess the secondary outcomes of stroke severity and post‐stroke sequelae in patient with AF and gout. A *P* value of less than .05 was considered statistically significant. STATA (IC‐15.1 version, STATA Corp) was used for the statistical analyses.

## 3 RESULTS

During the year 2016, we identified 3 844 057 admissions among patients with AF, of whom 240 875 patients had history of gout. Table 1 describes a comparison of baseline characteristics between patients with AF, with and without a history of gout. Patients with AF who had gout compared to those without gout had lower proportion of females (31.9% vs 48.4%, *P* < .001), Whites (73.6% vs 78.7%, *P* < .001) and Hispanics (3.6% vs 5.3%, *P* < .001). There was a higher proportion of Blacks (13.8% vs 8.3%, *P* < .001) and Asian/Pacific Islander (3.3% vs 1.8%, *P* < .001) among patients AF and gout compared to patients with AF without gout. There was no significant difference in the proportion of Native Americans. In terms of past medical illnesses, patients with AF and gout compared to without gout had higher prevalence of obesity (13.1% vs 9%, *P* < .001), DM (20.9% vs 19.1%, *P* < .001), CHF (11.7% vs 9.1%, *P* < .001), dyslipidemia (55.5% vs 46.4%, *P* < .001), CAD (47.8% vs 40.2%, *P* < .001), PVD (9.8% vs 7.6%, *P* < .001), and CKD/ESRD (57.2% vs 39.1%, *P* < .001). The prevalence of hypertension and tobacco smoking was lower in patients with AF and gout compared to AF with gout (31.9% vs 48.4% and 5.6% vs 9.1%, respectively, *P* < .001). There was no significant difference in age (75.5 ± 0.1 vs 75.6 ± 0.05 years, *P* = .162) between the two groups. The prevalence of a prior history of stroke or TIA was slightly higher in patients with AF and gout compared to AF patients without gout (12.5% vs 12.6%, *P* = .642), however, the difference did not reach statistical significance.

**TABLE 1:** Basal characteristics compared between patients with AF, with and without gout

Basal characteristics	AF^a^ without gout (n = 3 603 182)	AF^a^ with gout (n = 240 875)	*P* value
Mean age (years)	75.6 (±0.05)	75.5 (±0.1)	.162
Female	1 743 940 (48.4%)	76 839 (31.9%)	<.001
Race/ethnicity			
White	2 835 704 (78.7%)	177 284 (73.6%)	<.001
Black	299 064 (8.3%)	33 241 (13.8%)	<.001
Hispanic	190 969 (5.3%)	8672 (3.6%)	<.001
Asian/Pacific Islander	64 857 (1.8%)	7949 (3.3%)	<.001
Native American	10 810 (0.3%)	482 (0.2%)	.001
Other	187 365 (5.6%)	13 248 (5.5%)	.477
Hypertension	1 711 511 (47.5%)	78 284 (32.5%)	<.001
Obesity	324 286 (9%)	31 555 (13.1%)	<.001
Diabetes Mellitus	688 208 (19.1%)	50 102 (20.8%)	<.001
Congestive heart failure	327 890 (9.1%)	28 182 (11.7%)	<.001
Dyslipidemia	1 671 876 (46.4%)	133 686 (55.5%)	<.001
Coronary artery disease	1 448 479 (40.2%)	155 138 (47.8%)	<.001
Peripheral vascular disease	273 842 (7.6%)	23 606 (9.8%)	<.001
History of stroke or TIA^b^	450 398 (12.5%)	30 350 (12.6%)	.642
Tobacco smoking	327 890 (9.1%)	13 489 (5.6%)	<.001
Chronic kidney disease and ESRD^c^	1 149 415 (31.9%)	137 781 (57.2%)	<.001

^a^ AF, atrial fibrillation.

^b^ TIA, transient ischemic attack.

^c^ ESRD, end stage renal disease.

There were 159 440 patients with AF who had new stroke in our study, of them 7690 (4.8%) had gout. This constitutes an incidence of 3.2% (n = 7690) of new stroke among all patients with AF and gout (n = 233 185). The baseline characteristics of patients with AF and gout who had new stroke were compared with patients with AF and gout who did not have new stroke, (Table 2). Those who had new stroke were older (77.8 ± 0.26 vs 75.5 ± 0.08 years, *P* < .001), were more females (35.2% vs 31.2%, *P* = .001), were less White (74.9% vs 76.7%, *P* = .022), were more likely to have HTN (47.5% vs 32.0%, *P* < .001), dyslipidemia (64.2% vs 55.2%, *P* < .001), and prior history of stoke or TIA (17.2% vs 12.5%, *P* < .001). There was lower prevalence of CKD/ESRD (42.4% vs 57.8%, *P* < .001), CAD (41.9% vs 47.9%, *P* < .001), and obesity (9.4% vs 13.2%, *P* < .001) in patients who had new onset stroke. There was no difference in the prevalence of DM (20.6% vs 20.8%, *P* = .790) or tobacco smoking (5.9% vs 5.6%, *P* = .536) as shown in Table 2.

**TABLE 2:** Basal characteristics compared between patients with AF and gout who had a stroke and who did not have a stroke

Basal characteristics	AF^a^ with gout without stroke (n = 233 185)	AF^a^ with gout with new stroke (n = 7690)	*P* value
Mean age (years)	75.5 (±0.08)	77.8 (±0.26)	<.001
Female	72 754 (31.2%)	2707 (35.2%)	.007
Race/ethnicity			
White	178 853 (76.7%)	5760 (74.9%)	.022
Black	33 579 (14.4%)	1107 (14.4%)	
Hispanic	8628 (3.7%)	300 (3.9%)	
Asian/Pacific Islander	7928 (3.4%)	385 (5.0%)	
Native American	466 (0.2%)	15 (0.2%)	
Other	3731 (1.6%)	123 (1.6%)	
Hypertension	74 619 (32.0%)	3653 (47.5%)	<.001
Obesity	30 780 (13.2%)	723 (9.4%)	<.001
Diabetes Mellitus	48 502 (20.8%)	1584 (20.6%)	.790
Congestive heart failure	27 283 (11.7%)	823 (10.7%)	.227
Dyslipidemia	128 718 (55.2%)	4937 (64.2%)	<.001
Coronary artery disease	111 696 (47.9%)	3222 (41.9%)	<.001
Peripheral vascular disease	22 852 (9.8%)	638 (8.3%)	.056
History of stroke or TIA^b^	29 148 (12.5%)	1323 (17.2%)	<.001
Tobacco smoking	13 058 (5.6%)	454 (5.9%)	.536
Chronic kidney disease and ESRD^c^	134 781 (57.8%)	3261 (42.4%)	<.001

^a^ AF, atrial fibrillation.

^b^ TIA, transient ischemic attack.

^c^ ESRD, end stage renal disease.

The primary outcome was the risk of new stroke in patients with AF and gout. Patients with AF and gout compared to AF without gout had a higher risk of new stroke (unadjusted OR 2.07 [1.97‐2.19], *P* < .001). Although this association was attenuated after adjustment for confounders, the presence of gout in patients with AF remained independently associated with the risk of new stroke after adjustment for CHADS2 variables (OR 1.24 [1.16‐1.32], *P* < .001). We also adjusted for CHADS2VASC variables along with any other significantly different baseline characteristic variable that is not accounted for in the CHADS2VASC score between patients with AF and gout who had new stroke and those who did not have stroke (Table 2). These variables were CKD/ESRD, dyslipidemia, obesity, and race. The risk remained significantly elevated (OR 1.10 [1.01‐1.11], *P* = .041) as shown in Table 3.

**TABLE 3:** Unadjusted and adjusted odds ratio of new stroke in patients with atrial fibrillation and gout

	Odds ratio	95% Confidence interval	*P* value
Stroke risk (unadjusted)	2.07	1.97‐2.19	<.001
Stroke risk (adjusted to CHADS2 score)	1.24	1.16‐1.32	<.001
Stroke risk (adjusted to CHADS2VASC score, CKD/ESRD, dyslipidemia, obesity and race)	1.10	1.01‐1.11	.041

Abbreviation: CKD/ESRD, chronic kidney disease, end stage renal disease.

The association between gout and post‐stroke sequelae (secondary outcomes) among patients with AF is shown in Table 4. On multivariable logistic regression adjusted for CHADS2VASC variables, CKD/ESRD, dyslipidemia, obesity, and race, the presence of gout in patients with AF was not associated with a higher risk of all‐cause in‐hospital mortality (4.6% vs 5.6%, OR 0.79 [0.61‐1.03], *P* = .076), need for MV (2.9% vs 3.2%, OR 0.96 [0.71‐1.31], *P* = .376), need for PEG tube insertion (4.3% vs 5.2%, OR 0.89 [0.68‐1.16], *P* = .091), or discharge to a skilled nursing facility (73.7% vs 75.8%, OR 1.02 [0.95‐1.09], *P* = .122). The hospital LOS for stroke was slightly longer in patients with AF and gout compared to without gout (6.7 vs 6.2 days, *P* = .040).

**TABLE 4:** Post‐stroke sequalae outcomes: By multivariate linear regression adjusted for CHADS2VASC score variables in addition to chronic kidney disease, dyslipidemia, obesity, and race

	Stroke in AF^a^ with gout (n = 7690) vs AF without gout (n = 151 750)	Multivariable odds ratio (95% CI)	*P* value
All‐cause in‐hospital mortality, n (%)	355 (4.6%) vs 8525 (5.6%)	0.09 (0.61‐1.03)	.076
Hospital length of stay (days)	6.7 days vs 6.2 days	Coef. 0.36 (0.02‐0.71)	.040
Need for mechanical ventilation, n (%)	220 (2.9%) vs 4845 (3.2%)	0.96 (0.71‐1.31)	.376
PEG^b^ tube placement, n (%)	330 (4.3%) vs 7835 (5.2%)	0.89 (0.68‐1.16)	.091
Discharge to skilled nursing facility, n (%)	5670 (73.7%) vs 115 085 (75.8%)	1.02 (0.95‐1.09)	.122

^a^ AF, atrial fibrillation.

^b^ PEG, percutaneous endoscopic gastrostomy.

## 4 DISCUSSION

The current study focused on the association between gout and cardiovascular disease, specifically AF and stroke. Among patients with AF, presence of gout was associated with a higher risk of stroke not worse post‐stroke outcomes.

The relationship between gout and cardiovascular comorbid conditions is complex. Many cardiovascular illnesses predispose to gout and vice versa.^10^ Recent evidence has shown that different comorbid conditions such as cluster together in people with gout, such as obesity, DM, heart failure, dyslipidemia, CAD, PVD, COPD, OSA, and CKD/ESRD.^11^, ^12^, ^13^, ^14^, ^15^, ^16^, ^17^, ^18^ The current study is in line with these associations as shown in Table 1. Despite the fact that HTN is the most common risk factor for the development of AF, our results showed a paradoxically lower prevalence of HTN in the gout group compared to subjects without gout.^19^ This lower prevalence of HTN in subjects with AF and gout in our study may be due to the higher prevalence of other risk factors of AF, such as DM, CKD, and obesity, in subjects with gout resulting in a lower relative contribution of HTN to the development of AF as compared to subjects without gout.

Patients with AF and gout who had new stroke when compared to patients who did not have a stroke were older, had higher share of female gender, were more likely to have HTN, dyslipidemia, and history of stroke or TIA. But the same group did not have a higher prevalence of the other traditional risk factors of stroke in AF, such as CAD, PVD, and CHF, as shown in Table 2. One possible explanation is the different interaction between gout and the traditional risk factors of stroke as they cluster and accumulate in patients with AF. There is evidence that gout and hyperuricemia have lower magnitude of risk in predicting stroke in patients with higher CHADS2VASC score. Chao T‐F et al, found a higher risk of hyperuricemia in predicting stroke when CHADS2VASC score is zero while the risk became insignificant for patients with score ≥4.^20^ There was a lower prevalence of CKD/ESRD in the stroke group, this might be explained by the fact that this study included all stages of CKD where milder stages did not have a significantly elevated risk for stroke.

Our results show a higher risk of new stroke on admission in patients with AF and gout before and after adjustment to CHADS2 score, CHADS2VASC score, CKD/ESRD, dyslipidemia, obesity, and race (Table 3). It has been shown that hyperuricemia increases the risk of left atrial thrombus, which raises the possibility of cardioembolic events.^21^, ^22^, ^23^, ^24^ The association between stroke and gout in subjects with AF was dampened significantly by multivariable adjustment although gout remained significantly associated with stroke. The increased risk of stroke in patients with gout and AF appears to be predominantly mediated by a higher prevalence of traditional risk factors in those with gout although traditional risk factors may not fully account for this increased risk. The severity of the stroke did not seem to be significantly different between AF patients with or without gout in our study as evidenced by similar rates of all‐cause in‐hospital mortality as well the need for mechanical ventilation, PEG tube insertion, and discharge to skilled nursing facility. Whether this finding is due to the origin of the stroke in patients with gout and AF, cardioembolic vs large cranial vessels thrombosis, and how it reflects on post‐stroke sequalae remains unclear.

The current study enjoys the strength of its nationwide representation and large sample size. This study has several important limitations inherent to large administrative databases. First, we used inpatient diagnosis codes and there is a potential for misclassification of disease. Second, this is a retrospective observational study, which may be subject to traditional biases of observational studies such as selection bias. Third, the NIS does not provide accurate details about the specific type of AF whether it was valvular vs nonvalvular. It was also not possible to determine the home or discharge medications and adherence to guideline directed therapies from the database especially in terms of anticoagulation and gout medications. The uric acid level is not provided in this large administrative database which precluded studying the association between severity of gout and stroke. Finally, the severity of stroke in the current study was based on in‐hospital outcomes and discharge to a skilled nursing facility but was not based on actual imaging data.

### 4.1 Clinical implication

- In the current study, gout was an independent risk factor in predicting new stroke in patients with AF but did not predict worse stroke outcomes or severity.
- The current study adds to the limited but growing evidence on the role of gout and hyperuricemia in refining the risk stratification of stroke in patients with AF.

## 5 CONCLUSION

Presence of gout in patients with AF is associated with stroke but not poor in‐hospital outcomes. It remains to be seen if future prospective studies would further investigate the role of gout in predicting the risk of stroke in patients with AF and whether gout and hyperuricemia can be integrated into future risk assessment scores to improve the prediction of stroke in AF patients.

## CONFLICT OF INTEREST

SV: Research support: Department of Veterans Affairs, World Heart Federation, Tahir and Jooma Family Honorarium: American College of Cardiology (Associate Editor for Innovations, acc.org).

## Supporting information

Table S1
