# Supplementary material for: The risk and severity of stroke in patients with atrial fibrillation and gout: A National Representative Database study
Source: J Arrhythm. 2021 Jan 25;37(2):394–9. doi: 10.1002/joa3.12505 (PMC8021996; doi:10.1002/joa3.12505)
Supplement: Supplementary file 1 — Table S1 [file JOA3-37-394-s001.docx]

**Supplement:**

**Table 1:** International Classification of Diseases, Tenth Revision, Clinical Modification codes (ICD‐10‐CM) used in the study:

| Diagnosis | ICD‐10‐CM code |
| --- | --- |
| Atrial fibrillation | I48.0 I48.1 I48.2 I48.91 |
| Gout (including all joints and chronicity) | M10.00 M10.01 M10.011 M10.012 M10.019 M10.021 M10.022 M10.029 M10.031 M10.032 M10.032 M10.039 M10.041 M10.042 M10.049 M10.051 M10.052 M10.059 M10.061 M10.062 M10.069 M10.071 M10.072 M10.079 M10.11 M10.111 M10.112 M10.119 M10.121 M10.0122 M10.129 M10.131 M10.132 M10.139 M10.141 M10.142 M10.149 M10.151 M10.152 M10.159  M10.161 M10.162 M10.169 M10.171 M10.172 M10.179 M10.18 M10.19 M10.20 M10.211 M10.212 M10.219 M10.221 M10.222 M10.229 M10.231 M10.232 M10.239 M10.241 M10.242 M10.242 M10.249 M10.251 M10.252 M10.259 M10.261 M10.262 M10.269 M10.271 M10.272 M10.279 M10.28 M10.29 M10.30 M10.311 M10.321 M10.322 M10.329 M10.331 M10.332 M10.339 M10.341 M10.342 M10.349 M10.351 M10.352 M10.359 M10.361 M10.362 M10.369 M10.371 M10.372 M10.379 M10.411 M10.412 M10.419 M10.421 M10.422 M10.429 M10.431 M10.439 M10.432 M10.441 M10.442 M10.449 M10.45 M10.452 M10.459 M10.461 M10.462 M10.469 M10.471 M10.472 M10.479 M10.49 M10.48 M10.9 |
| Cerebrovascular accident including all cranial arteries (excluding hemorrhagic) | I63.0 I69.31 I69.310 I69.311 I69.312 I69.313 I69.314 I69.315 I69.319 I69.32 I69.318 I69.320 I69.321 I69.322 I69.323 I69.328 I69.33 I69.330 I69.331 I69.332 I69.333 I69.334 I69.339 I69.34 I69.341 I69.342 I69.343 I69.344 I69.349 I69.35 I69.351 I69.352 I69.353 I69.354 I69.359 I69.36 I69.361 I69.362 I69.363 I69.364 I69.365 I69.369 I69.39 I69.390 I69.391 I69.392 I69.393 I69.398 I69.8 I69.80 I69.81 I69.810 I69.811 I69.812 I69.813 I69.814 I69.815 I69.818 I69.819 I69.82 I69.820 I69.821 I69.822 I69.828 I69.83 I69.831 I69.832 I69.833 I69.834 I69.839 I69.84 I69.841 I69.842 I69.84 I69.844 I69.84 I69.85 I69.851 I69.852 I69.853 I69.854 I69.859 I69.86 I69.861 I69.862 I69.863 I69.864 I69.865 I69.869 I69.89 I69.890 I69.891 I69.892 I69.893 I69.898 I69.9 I69.9 I69.90 I69.91 I69.910 I69.911 I69.912 I69.913 I69.914 I69.915 I69.918 I69.919 I69.92 I69.920 I69.921 I69.922 I69.923 I69.928 I69.93 I69.931 I69.932 I69.933 I69.934 I69.939 I69.94 I69.941 I69.942 I69.943 I69.944 I69.949 I69.95 I69.951 I69.952 I69.953 I69.954 I69.959 I69.96 I69.961 I69.962 I69.963 I69.964 I69.969 I69.965 I69.99 I69.990 I69.991 I69.992 I69.993 I69.998 G45.0 G45.1 G45.2 G45.3 G45.8 G45.8 G45.5 G45.9 G46.0 G46.1 I63.10 I63.111 I63.112 I63.113 I63.119 I63.131 I63.132 I63.133 I63.139 I63.20 I63.211 I63.212 I63.213 I63.219 I63.22 I63.231 I63.232 I63.233 I63.239 I63.29 I63.40 I63.411 I63.412  I63.4113 I63.419 I63.421 I63.422 I63.423 I63.429 I63.431 I63.432 I63.433 I63.439 I63.441 I63.442 I63.443 I63.449 I63.50 I63.511 I63.512 I63.519 I63.521 I63.522 I63.523 I63.529 I63.531 I63.532 I63.539 I63.541 I63.542 I63.543 I63.549 I6359 I63.8 I63.9 |
| Hypertension | I10 |
| Diabetes Mellitus | E11.9 |
| Congestive heart failure | I50.30 I50.32 I50.20 |
| History of stroke | Z86.73 |
| Tobacco smoking | Z72.0 F17.210 F17.200 |
| Dyslipidemia | E78.5 E7800 E78.2 E78.49 |
| Peripheral vascular disease | I73.9 |
| Coronary artery disease | I25.2 I25.10 |
| Chronic kidney disease and end stage renal disease | N18.1 N18.2 N18.3 N18.4 N18.5 N18.6 N18.9 |
| Mechanical ventilation and intubation | 0BH17EZ 5A19054 5A1935Z 5A1945Z 5A09357 |
| Percutaneous gastrostomy tube | 0DH60UZ 0DH63UZ 0DH64UZ 0DH67UZ 0DH68UZ 0DH80UZ 0DH83UZ 0DH84UZ 0DH87UZ 0DH88UZ 0DH90UZ 0DH93UZ 0DH94UZ 0DH97UZ 0DH98UZ 0DHA0UZ 0DHA3UZ 0DHA4UZ 0DHA7UZ 0DHA8UZ 0DHB0UZ 0DHB3UZ 0DHB4UZ 0DHB7UZ 0DHB8UZ |
